# Evaluation of intra-osseous jaw lesion diagnosis using fine needle aspiration cytology among Indian patients

**DOI:** 10.6026/9732063002001789

**Published:** 2024-12-31

**Authors:** Nisha Kumari, Smita Kumari Gupta, Nishat Ahmad, Sunil Kumar Mahto, Moazzam Jawaid

**Affiliations:** 1 Department of Pathology, Rajendra Institute of Medical Sciences (RIMS), Ranchi, Jharkhand, India; 2 Department of Oral Medicine and Radiology, Sarjug Dental College and Hospital, Darbhanga, Bihar, India

**Keywords:** Fine needle aspiration cytology (FNAC), bone lesions, intra-osseosous

## Abstract

Fine needle aspiration cytology (FNAC) has historically been used to diagnose thyroid cancers, growths on the neck, salivary glands and other ailments. Therefore, it is of interest to evaluate efficacy of FNAC in the diagnosis of intra-osseous jaw pathologies among Indian patients. Diagnosis obtained through FNAC was correlated with histopathological examination in 42 cases with an accuracy of 84%. The sensitivity of FNAC in diagnosing bone lesions was 80% and the specificity was 88%. The positive predictive value was 86.9%and negative predictive value was 81.4%. Thus, the efficiency of FNAC in the diagnosis of lesions in the intra-osseous jaw among Indian patients is reported.

## Background:

A connective tissue that provides the body with mechanical reinforcement is bone. Its intricate development, growth and maintenance leave it vulnerable to neoplastic, congenital, circulatory and inflammatory disorders [1, 2]. There are many different types of bone disorders, so attempts have been made to provide a straightforward, trustworthy and accurate diagnosis method that will make patient care easier. For more than a century, the foundation for detecting bone lesions has been diagnostic histology [3, 4]. These days, the identification of bone lesions is becoming more and more common by fine needle aspiration cytology (FNAC). For a large number of patients, the FNAC result in conjunction with the assessment of clinical as well as radiologic data has been adequate to guide choices regarding treatment [5-6].

Prior to receiving the final therapy, biopsy has only been required in a small percentage of individuals [7, 8]. FNAC by itself is a reliable diagnostic tool for a large number of distinct bone cancers, both benign and malignant [9, 10]. In many centers, cytodiagnosis of bone abnormalities and fine needle biopsy (FNB) are already standard practices. A genuinely thin needle (22-23 gauges) cannot pass through undamaged corticated bone or sclerotic pathologies, although it can frequently pass through partially disintegrated or "moth-eaten" cortical bone [11, 12, 13-14]. Drilling through undamaged cortical bone and inserting a tiny needle within the lesion via the drilled canal are now feasible thanks to new technologies like the Bone Biopsy device, coaxial biopsy equipment utilizing an eccentric drill [15, 16, 17-18]. Compared to open biopsy, FNB is less invasive on bone, allows for many samples without any problems and leaves no scar. Infection is not a concern if basic sterility is maintained. FNB is an easy, quick and affordable outpatient surgery [19, 20- 21]. Its main goals include not only determining the morphologic screening of benignity or malignancy, looking into possible bone secondary tumors and occasionally taking material samples from osteolytic lesions that are radiologically presumed of being osteomyelitis to establish a bacteriologic diagnosis [22, 23, 24, 25]. If at all possible, it also seeks to substitute open or coarse needle biopsy in the recognition of primary bone tumors prior to treatment. The radiological assessment, FNAC and clinical findings make up the diagnostic triad.

A biopsy is necessary in addition to standard X-rays [12, 13, 14-15]. Large lesions are easily aspirable without image assistance, but many lesions may gain advantage from image guiding to increase the diagnostic value from a subsequent microscopic inspection and enhance lesion targeting accuracy [10-16]. Traditionally, thyroid tumors, growths of the neck as well as salivary glands and other conditions are diagnosed by FNAC. There is insufficient evidence to support the effectiveness of FNAC in the diagnosis and management of intra-osseous jaw bone pathologies [17, 18-19]. Since intra-osseous jaw lesions are close to tooth apices along with neurovascular bundles, diagnosing them can be challenging [20, 21, 22- 23]. Instead of having an open biopsy to obtain a conclusive histological diagnosis, many patients are routinely followed up for extended periods of time in order to search for sequential radiographic changes [21, 22, 23, 24-25]. Consequently, important or dangerous diagnoses are occasionally postponed. If a proper link with clinico-radiological results is made, FNAC can provide the doctor with a conservative solution to more invasive treatments like open biopsy. It is necessary to determine the efficacy of FNAC in the diagnosis of intra-osseous jaw lesions. Therefore, it is of interest to assess the efficacy of FNAC in the diagnosis of intra-osseous jaw lesions among Indian patients.

## Methods and Materials:

The present study was conducted in the Department of Pathology, Rajendra Institute of Medical Sciences and Ranchi. Ethical clearance was obtained from the institutional ethical committee of Rajendra Institute of Medical Sciences and Ranchi with reference number 40, IAEC/2 RC RIMS, RANCHI, dated 20-02-2018.

## Inclusion criteria and exclusion criteria:

The patient complaining with palpable bony mass lesion, bone pain, pathological fracture of all ages and both genders were included in study. While patients with history of previous diagnosed case and receiving therapy, history of recurrence of lesion were excluded from the study.

## Sample size:

A total of 54 cases of bony lesion were studied fulfilling the inclusion and exclusion criteria.

## Methodology:

The information collected from the patients and their case records were kept confidential and the patients were given full freedom to withdraw at any point during the study period. To reach a cytological diagnosis following points were taken into account.

## Patient selection:

After detailed disease history and thorough clinical examination the bony disease process was localized and clearly defined by clinical and radiological imaging technique like X-ray, C.T scan and M.R.I. Possible risk factors like coagulation disorders and uncontrolled diabetes were taken into consideration.

## Procedure of FNAC:

## Equipment:

Standard 18-22 gauge needle were used for superficial palpable bony lesions with cortical erosion. Good quality 10 ml disposable syringes were used to produce negative pressure. The holder leaves one hand free to immobilize and feel the target lesion and this allows more precision in placing the needle. Small sterile containers with tight lid containing physiological saline were kept ready to rinse needle and syringes if culture was needed. Anticoagulant containing vials were used for haemorrhagic aspirates. Clean, dry, grease free, labelled slides were used. The smear was smeared between two standard microscopic slides. Fixatives - (70-90%) ethanol kept in coplin jar were used. Stains used were Leishman - Giemsa stain, Haematoxylin - Eosin stain and Routine Papanicolaou stain. Binocular inclined microscopes were used to assess the adequacy of sample. Skin disinfectant, sterile cotton swabs, spirit, cover slips, latex gloves, face mask were kept ready at the time of aspiration.

## Patient preparation:

Most FNAC were carried out with patient lying supine on an examination table, placed in such a way that there is easy access to the site of lesion. Surgical skin disinfectant (Savlon, Betadine), a fenestrated sterile cloth and sterile surgical gloves were used. Anesthesia was not required in most of the patients. Local anesthesia to suppress periosteal pain was used in few cases in conjunction with CT guidance and where several needle passes were required to obtain adequate sample.

## FNAC procedure:

Syringe is held by one hand and leaving the other hand free to feel and fix the target. Occasional radiological imaging technique to guide deep aspirations was also done. To increase the yield, the needle was moved back and forth within the lesion with the negative pressure maintained. The negative pressure does not tear the cells from the tissue but merely hold the tissue against the sharp cutting edges of the needle. Many passes of the needle were sometimes required to obtain sufficient material. Admixture with blood tends to be less when needle was moved along the same track rather than in various directions. The negative pressure was released before the needle was withdrawn, so that the material in the needle and hub would not be sucked into the cylinder of the syringe.

## Processing the sample:

The sample contained in the needle was expelled on the clean and dry microscopy slide using air in the syringe, taking care to avoid splashing. Sometimes the best part of the sample was hub of the needle, which could not be expelled by blowing of air, in such cases sample was retrieved by aspiration with other needle or by picking it with a fine wooden stick.

## Direct smearing:

The 'Dry' aspirate consisted of numerous cells suspended in the small amount of tissue fluid which was evenly spread on a standard glass slide moving the slide steadily and evenly over the specimen slide while exerting light pressure. The 'Wet' aspirated consisted of smaller number of suspended in the fluid or blood. Thin smears of 'Dry' aspirate dry almost instantaneously and drying artifacts are difficult to avoid when smears are wet fixed.

## Indirect smearing:

Thin fluid aspirated was best processed by centrifugation in order to concentrate the cells and separate them from fluid. After removing the excess supernatant, the cells were re-suspended and then smeared on the glass slides as dry and wet fixed smears.

## Fixation and staining:

Two fundamentally different methods of fixative and staining are used in the FNAC: Air drying followed by staining with LG stain containing leishaman and Giemsa. Alcohol fixation followed by (H & E) Haematoxylin and Eosin / Papanicolaou stain.

## Histopathological examination (HPE):

The association between histopathology and excision, curettage and intraoral biopsy was determined and the FNAC's diagnostic accuracy was computed.

## Data entry and analysis:

Data obtained were entered in Microsoft Excel - 2007 after proper template generation and analysis was done using SPSS software version 20. In the present study, analyzed data was expressed in the terms of frequency and percentages. Appropriate tests were applied to compare the categorical data and significance was taken as p-value < 0.05.

## Results:

In the present study, we found that 50 samples were adequate for diagnosis out of 54 cases and adequacy of smears was 92.5%. Diagnosis obtained through FNAC was correlated with histopathological examination in 42 cases having an accuracy of 84%.18 (36%) cases were of benign neoplastic lesions according to FNAC evaluation. 14 (28%) cases were diagnosed as malignant lesion. 8 (16%) were benign non neoplastic. 7 (14%) cases were inflammatory lesion according to FNAC (Table 1). In benign lesions, on analysing the concordance between FNAC diagnosis and histopathological diagnosis, it was found that 1 case out of 5 cases of ABC (20%), 1 case out of 3 cases of SBC (33.33%), 1 case out of 2 cases of Osteoid osteoma (OO) (50%) had diagnosis non similar to that observed in FNAC. Rest cases of benign lesions had diagnosis in concordance with FNAC. The findings were statistically significant (p=0.02) (Table 2). In malignant lesions, it was observed that 1 case out of 6 cases (16.66%) of osteosarcoma was found to have histopathological diagnosis different from that observed in FNAC evaluation. Similarly, 1 case out of 5 cases (20%) of Ewing sarcoma was found to have histopathological diagnosis different from that observed in FNAC evaluation. All cases of malignant osteo-clastoma and chondrosarcoma had histopathological diagnosis similar to FNAC diagnosis. The findings were significant statistically (p=0.01) (Table 3). On comparing histopathological diagnosis with FNAC diagnosis in non-neoplastic inflammatory lesions, it was observed that 1 case out of 4 cases (25%) of chronic osteomyelitis had histopathological diagnosis different from FNAC diagnosis. 1 case out of 3 cases (33.33%) of tubercular osteomyelitis had histopathological diagnosis different from FNAC diagnosis. The findings were significant statistically (p-0.01) (Table 4). Finally, overall sensitivity of FNAC (cytological diagnosis) in diagnosing bone lesions was 80%. This high sensitivity indicates that test can be used for excluding or ruling out a condition when it was negative .The specificity was 88%. This high specificity indicates that test can be used for including or ruling in a condition when it was positive. The positive predictive value was 86.9%; the more likely the disease was present with positive test finding. The negative predictive value was 81.4%, the more likely the disease was absent with a negative test finding (Table 5).

## Discussion:

Acceptance of FNAC of bone as a diagnostic technique has been impeded by the inability to obtain adequate smears. The rate of adequacy of samples ranges from 66% to 100% in the various studies [6, 25]. In the present study, we found that 50 samples were adequate for diagnosis out of 54 cases and adequacy of smears was 92.5% which is similar as in the different studies. A research found that adequacy of samples was 95.3%. Similarly, another research observed that 90.47% was the adequacy of the samples [6, 25]. In few investigations adequacy of sample was found out to be 69% and 66% [18-21]. It was slightly lower than the adequacy of sample observed in our study. In the present study, age range lied between 4 years to 60 years. Maximum no of cases diagnosed as a bone tumours belonged to age group between 11-20 years which were 20 cases out of 47. Least no of cases have seen between age group 41- 60 years. This clearly states that bony lesions especially primary neoplasm tends to occurs at early age groups. This is in concordance with most of the studies. Literature found that most common age group was 21-30 years [6, 25]. In our study, 18 (36%) cases were of benign neoplastic lesions according to FNAC evaluation. 14 (28%) cases were diagnosed as malignant lesion. 8 (16%) were benign non neoplastic. 7 (14%) cases were inflammatory lesion according to FNAC. This is in concordance with most of the studies. [6, 25]. In the present study the accuracy of FNAC to diagnose true benign and malignant neoplastic lesion was found to be 84%. An accuracy of various studies ranged from 71% to 95.92%. Therefore, accuracy of the present study is in concordance with most of the studies like where the accuracy was 95%, 87.8% and 90.5% [20-25]. The sensitivity was 92%, specificity was 99%, positive predictive value was 99%and negative predictive value was 91% [24-25]. Similarly, the sensitivity was 95%, specificity was 94% in another research [6-9]. In another research, the sensitivity was 96.66%, specificity was 95.23%, positive predictive value was 97.75%and negative predictive value was 96% [6, 25]. After analysing FNAC data from 23 cases of radiolucent jaw lesions, some investigators came to the conclusion that FNAC is an effective method for separating benign from malignant jaw lesions. The use of narrow needles for aspiration is made possible by the weakening or degeneration of cortical bone [4, 6].

## Limitations of study:

The primary obstacles to a conclusive diagnosis in cases of discrepancy were non-representative sample, nonspecific morphological abnormalities on cytosmears and insufficient architectural context in the FNAC data. For example, analysing the lining cell features on FNAC is not practical to differentiate a dentigerous cyst with an odontogenic keratocyst.

## Conclusion:

The efficiency of FNAC in the diagnosis of lesions in the intra-osseous jaw among Indian patients is reported. Thus, FNAC is useful in diagnosing intra-osseous tumors of jaws, particularly in separating inflammatory from neoplastic lesions among Indian population.

## Figures and Tables

**Table 1:** Distribution of study participants according to type of lesions according to FNAC

	**Benign neoplastic**	**Benign non-neoplastic**	**Inadequate**	**Inflammatory**	**Malignant**
Number (n)	18	8	3	7	14
Percentage (%)	36	16	6	14	28

**Table 2:** Histopathological diagnosis and FNAC diagnosis in benign lesions (n=26)

	**ABC**	**SBC**	**CB**	**EC**	**OB**	**OCN**	**OC**	**OO**	**p-value**
No of cases according to FNACn (%)	5(19.23)	3(11.53)	1(3.87)	2(7.70)	1(3.87)	1(3.87)	1 (3.87)	2 (7.70)	0.02*
No of cases according to Histopathology examinationn (%)	4(15.24)	2(7.70)	1(3.87)	2(7.70)	1(3.87)	1(3.87)	1(3.87)	1(3.87)	
No of cases having dissimilar diagnosis according to FNAC and histopathologyn (%)	1(20%)	1 (33.3%)	0	0	0	0	0	1(50%)	
Aneurysmal bone cyst (ABC),
Simple bone cyst (SBC),
Chondroblastoma (CB),
Enchondroma (EC),
Osteoblastoma (OB),
Osteochondroma (OCN),
Osteoclastoma (OC),
Osteoid osteoma (OO)

**Table 3:** Histopathological diagnosis and FNAC diagnosis in malignant lesions (n=14)

	**Osteosarcoma**	**Malignant osteoclastoma**	**Ewing's sarcoma**	**Chondrosarcoma**	**p-value**
No of cases according to FNAC	6 (33.34)	2 (1.12)	5 (2.78)	1 (0.56)	0.01*
No of cases according to Histopathology examination n (%)	5 (2.78)	2 (1.12)	4 (2.23)	1 (0.56)	
No of cases having dissimilar diagnosis according to FNAC and histopathology n (%)	1 (16.67)	0	1 (20)	0	

**Table 4:** Histopathological diagnosis and FNAC diagnosis in non-neoplastic inflammatory lesions (n=7)

	**Chronic osteomyelitis**	**Tubercular osteomyelitis**	**p-value**
No of cases according to FNAC n (%)	4 (57.14%)	3 (42.85%)	0.01*
No of cases according to Histopathology examination n (%)	3 (42.85%)	2 (28.57%)	
No of cases having dissimilar diagnosis according to FNAC and histopathology n (%)	1 (25%)	1 (33.33%)	

**Table 5:** Parameters for diagnostic accuracy of FNAC in bone lesions

**Parameters**	**Percentage**
Sensitivity	80%
Specificity	88%
Positive predictive value	86.90%
Negative predictive value	81.40%
